# Detection of congenital heart disease by neonatologist performed cardiac ultrasound in preterm infants

**DOI:** 10.1038/s41372-024-02065-4

**Published:** 2024-07-23

**Authors:** Bradley MacDonald, Deane Yim, James Ramsay, Andrew Gill

**Affiliations:** 1 Department of Cardiology, Perth Children’s Hospital, Perth, WA Australia; 2 Cardiovascular Epidemiology Research Center, School of Population and Global Health, University of Western Australia, Perth, WA Australia; 3 Healthy Skin and ARF Prevention, Telethon Kid’s Institute, Perth, WA Australia; 4 Neonatal Intensive Care Unit, King Edward’s Hospital for Women, Perth, WA Australia; 5 School of Medicine, University of Western Australia, Perth, WA Australia

**Keywords:** Congenital heart defects, Medical imaging

## Abstract

**Objective:**

We aimed to assess the frequency of de novo congenital heart disease (CHD) detection via neonatologist-performed cardiac ultrasounds (NPCU) in premature infants born at <30 weeks of gestation.

**Study design:**

In this cross-sectional study (2004–2023) clinicians completing NPCU flagged de novo suspected CHD. All flagged NPCUs were cross-checked with cardiologists to confirm CHD diagnosis.

**Results:**

There were 2088 out of 3739 infants (56%) with at least one NPCU; 294 (14%) with cardiology referral. CHD diagnosis was confirmed in 109 of the 2088 (5.2%) infants. All major and critical CHD on NPCU imaging were suspected during NPCU and had prompt referral to the cardiology department.

**Conclusion:**

De novo presentation of significant CHD continues to occur in the preterm population, emphasizing the need for recognizing CHD during NPCU. Optimizing NPCU training may benefit patients with early cardiology referral and review.

## Introduction

Congenital heart disease (CHD) is the most common birth defect, occurring in approximately 1%of live births, with approximately 25% of these being defined as critical CHD [1, 2]. The nature of CHD often has a profound influence on the lives of patients. In the first year, significant morbidity and mortality rates were associated with certain types of CHD [3]. Delayed referral may increase the risk of death and serious lifelong morbidity [4]. Therefore, it is imperative for CHD, unable to be diagnosed prenatally, to be detected as early as possible by the treating team. Assisting our cardiology colleagues, with early referral when CHD is suspected, is imperative. The early detection of CHD has a measurable effect on CHD prevalence [5].

The use of neonatologist-performed cardiac ultrasound (NPCU) has increased over the past few decades [6]. In Australia and New Zealand, over 40% of neonatal intensive care units (NICU) perform NPCU, which is attributable to an improved advanced skill set of the performing clinician and readily available echocardiography machines and platforms [7, 8]. This has been driven by the clinical need for echocardiography within a critical population and is utilized for prompt hemodynamic assessment, such as in the setting of a patent ductus arteriosus (PDA) assessment, systemic hypotension, and hypoxic respiratory failure [9]. The focus of NPCU on CHD varies between centers and must be guided by cardiologists who ultimately diagnose and manage patients [10]. In addition, NPCU are most frequently undertaken in premature infants, where the risk of CHD may be higher and where early diagnosis may significantly influence their clinical course. Our service aimed to assess the utility of NPCU in the detection of critical and major CHD in extreme prematurity. Our secondary objectives include a review of the nature of CHD not detected by the NPCU, as well as the assessment of CHD detection in the NPCU in infants of all gestations seen by our service.

We have observed significant advances in fetal screening for CHD, including improved fetal echocardiography scanning and the addition of pulse oximetry screening. We aimed to assess the utility of NPCU in the detection of critical and major CHD in extreme prematurity. Our secondary objectives include a review of the nature of CHD not detected by the NPCU, as well as the assessment of CHD detection in the NPCU in infants of all gestations seen by our service.

## Material and methods

### Setting

King Edward’s Memorial Hospital for Children is the primary tertiary perinatal center in Western Australia (WA) servicing all significantly unwell infants born in the state. Author AG introduced a formal training program in NPCU in 2003. This program was developed in conjunction with pediatric cardiologists and emphasizes the importance of defining anatomy in addition to hemodynamic assessment. The training program is affiliated with the Australian Society of Ultrasound in Medicine Certificate in Clinician Performed Ultrasound, which requires mandatory training followed by supervised cardiac ultrasounds and case logging of twenty-five specific cardiac scans. All NPCU, in our center, are either completed by, or supervised and reviewed by, clinicians accredited with the Certificate in Clinician Performed Ultrasound. At the outset, a formal electronic reporting system was introduced. Infants with suspected cardiac anomalies were referred to the WA Pediatric Cardiology Team who continue to collaborate closely with neonatologists.

The Perth Children’s Hospital Cardiology Center is the only tertiary pediatric cardiology center in the state and cares for most CHD patients in the jurisdiction. The center also provides fetal screening. Pediatric cardiology data is recorded in an electronic database (Synapse^TM^ Cardiovascular Client V4.0.4, Fujifilm Medical Systems USA) as well as a clinical cardiology database (Cardiobase^TM^, Version 8.1.44.10, Derby, UK).

### Cases

Patients with suspected CHD were identified from neonatal electronic records and cross-checked with a formal pediatric cardiology scan. As we sought to identify only those cases selected de novo by the NPCU, infants with prenatally expected CHD, those previously scanned and identified by cardiology, or with syndromes suggestive of CHD were excluded. We restricted the analysis to infants less than 30 weeks of gestational age, as these infants were most likely to receive NPCU. Infants greater than 30 weeks of gestation were more likely to undergo cardiac referral for clinical symptoms suggestive of CHD, rather than hemodynamic assessment. In our center, patients with PDA at discharge are referred to cardiology, even though they do not meet the formal definition of CHD based on age ( > 3 months corrected age). As such, we excluded patients with PDA identified on the NPCU for cardiology referral, as few of them met CHD criteria.

### Definitions

CHD was defined in accordance with the International Pediatric Congenital Cardiac Code [11]. In this study, critical CHD was defined by the need for surgical or catheter-based intervention at less than 30 days of life (or death prior to surgery that would otherwise be critical), major as having intervention between 30 days of life and less than 12 months of life, and non-major as having either no intervention or required intervention at any other point during the study period, including following NICU discharge. The maximum age of follow up within pediatric cardiology services is 16 years of age for CHD detection. Prematurity, as a term, is used to delineate infants born prior to 30 weeks of gestation at birth for the purpose of this study.

The term Neonatologist Performed Cardiac Ultrasound (NPCU) was used here to describe cardiac ultrasound performed at the bedside by trained neonatologists to assess cardiac function and transitional circulation within the context of the Australian training system, which includes some initial anatomical assessment. This definition may be interchangeable to some degree with that of targeted neonatologist echocardiograms, or TnECHO [12], as well as other terms such as neonatologist-performed echocardiography or NPE [6, 13]. We have refrained from using the term ‘echocardiography’ to delineate it clearly from cardiologist performed echocardiograms. The term NPCU is used instead of cardiac point of care ultrasound, or POCUS, as this term often refers to non-imaging specialist practitioners, extend beyond cardiac assessment alone and may overlook the specific skills, training and supervision required for NPCU in our setting [14–16].

Inclusion criteria included patients with NPCU under 30 weeks gestation. Suspected CHD on NPCU was referred to the cardiology department with final CHD diagnosis determined by cardiologist assessment. Accurate diagnosis was ensured by reviewing most recent echocardiogram of patients allowing delineation of lesions less likely to be detected within early life and in premature infants (for example patent foramen ovale (PFO) versus atrial septal defect (ASD)).To determine the accuracy of NPCU, the cohort was subdivided into a *flagged* group, defined as patients flagged for CHD by NPCU with diagnosis confirmed with a formal echocardiogram, and the *other* group, who were not referred to cardiology based on NPCU but by other means (clinical signs, follow-up review, etc.). In addition to the critical/major CHD requiring intervention, we audited non-major CHD diagnoses.

### Statistical analysis

Demographic and clinical information are presented as proportions with percentages or medians with interquartile ranges. Odds ratios with confidence intervals are used to reflect the likelihood of NPCU in screening for CHD within the cohort. All statistical analyses were performed using R, Version 1.4.1717 (R Foundation for Statistical Computing, Vienna, Austria) [17].

## Results

There were 3739 preterm infants, <30 weeks gestation, admitted to our service during the study period with2088 (56%) infants undergoing NPCU. In total, 2886 infants received NPCU between 2004 and 2023, of whom 669 were excluded: with a majority being greater than 30 weeks gestation and a further 129 infants with PDA alone. Sixteen patients with syndromic findings associated with CHD and 64 with suspected CHD on prenatal imaging were also excluded.

**Fig. 1:**
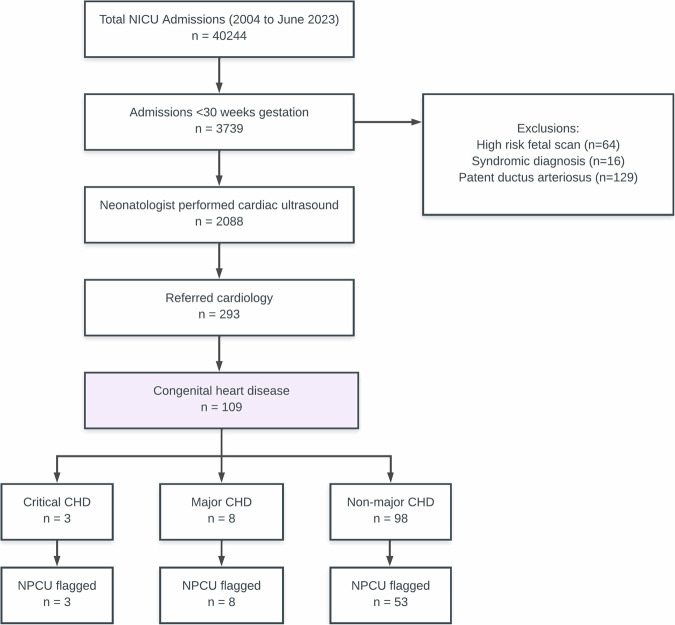
Neonatologist-performed cardiac ultrasound (NPCU) patients with subsequent cardiology-led echocardiogram and formal diagnosis of congenital heart disease (CHD). CHD congenital heart disease, NPCU neonatologist performed cardiac ultrasound, NICU neonatal intensive care unit.

Of the remaining infants, 293 (14%) were referred for a formal cardiology echocardiogram, of whom 12 (<1%) had CHD requiring cardiac intervention. There were three patients with critical CHD, eight had major CHD, and one had non-major CHD that required cardiac intervention. The flow diagram of the study population is demonstrated in Fig. 1.

Of the 293 patients referred to cardiologists from the NPCU, there were 109 (38%) with CHD. In total, 64 (58% of those with CHD) were *flagged* and referred to cardiology after suspicion of CHD during NPCU, with 45 (42%) in the *other* group referred to cardiology via clinical means later. The patients’ baseline characteristics are shown in Table 1.

**Table 1:** Baseline characteristics of patients undergoing NPCU with formal CHD diagnosis by referral from NPCU or referral by other pathways.

Demographics	Flagged by NPCU *n* = 64	Flagged by other means *n* = 45
Gestational age (weeks)	26.3 (3.2)	26.3 (2.0)
Sex, female	33 (51)	24 (52)
Critical CHD	3 (5)	0 (0)
Major CHD	8 (12)	0 (0)
Non-Major CHD	53 (83)	45 (100)
Cardiac surgery (initial)		
Coarctation repair	3 (5)	0 (0)
Balloon Pulmonary Valvotomy	1 (2)	1 (2)
Tetralogy of Fallot repair	2 (3)	0 (0)
Ventricular septal defect repair	3 (5)	0 (0)
Death prior to surgery	2 (3)	0 (0)

The NPCU *flagged* 183 cases that did not have CHD with reliable exclusion of 1794 cases (true negatives). Flagged cases, without CHD on formal cardiology assessment, were those assessed for transitional circulation or critical illness via NPCU. Only one surgical procedure, in a non-major CHD diagnosis, was detected in the *non-flagged* group. Eleven patients in the flagged group had critical or major CHD undergoing cardiac surgery or procedures in the *flagged* group. Other demographic features were comparable between the groups, including location in a metropolitan area (35 patients in the *other* group and thirty-five in the *flagged* group), although twenty-three patients had no recorded postcodes. Gestational age and sex distributions were comparable (Table 1).

CHD flagged by NPCU included ASD (17%), pulmonary stenosis (PS) (28%), and ventricular septal defects (VSD) (39%) (Table 2). The most frequent cardiac lesions not flagged by NPCU also included ASD (37%), PS (50%), and VSD (11%). All critical or major lesions that required immediate intervention were flagged by the NPCU. One patient in the false-negative group had CHD classified as non-major, with intervention required after the first year of life for balloon pulmonary valvuloplasty in the context of pulmonary stenosis. Two patients with critical CHD died without surgical intervention due to a combination of CHD and prematurity. One patient was diagnosed with pulmonary atresia that did not receive intervention and the other had tetralogy of Fallot; however, both patients had complications associated with prematurity contributing to outcome.

**Table 2:** Congenital heart disease (CHD) diagnosis in the flagged and other groups of patients undergoing neonatologist performed cardiac ultrasound.

	All CHD		Critical/major CHD		Non-major	
CHD Diagnosis	Flagged	Other	Flagged^a^	Other	Flagged	Other
Total	64	45	11	0	53	45
Atrial septal defect	11 (17)	17 (37)	0 (0)	0 (0)	11 (21)	17 (37)
Bicuspid aortic valve	0 (0)	1 (2)	0 (0)	0 (0)	0	1 (2)
Coarctation/hypoplastic aortic arch	4 (6)	0 (0)	3 (27)	0 (0)	1 (2)	0 (0)
Left ventricular outflow tract obstruction	1 (1)	0 (0)	0 (0)	0 (0)	1 (2)	0 (0)
Pulmonary atresia	1 (1)	0 (0)	1 (1)^a^	0 (0)	0 (0)	0 (0)
Pulmonary stenosis	18 (28)	22 (50)	1 (9)	0 (0)	17 (32)	22 (49)
Tetralogy of Fallot	4 (6)	0 (0)	3 (27)^a^	0 (0)	1 (2)	0 (0)
Ventricular septal defect	25 (39)	5 (11)	3 (27)	0 (0)	22 (41)	5 (11)

^a^Includes patients who died having not received surgery.

The odds ratio of CHD diagnosis if flagged during NPCU in prematurity (<30 weeks gestation) was 13.64 (9.07, 20.52), with a positive predictive value of 26% (sensitivity 58%, specificity 91%). If non-major CHD is excluded from analysis, then the sensitivity for NPCU for major CHD, including critical disease, is 100% with a specificity 90.74%. At greater than 30 weeks gestation the odds ratio is 15.33 (7.71, 30.48) and for the whole cohort is 14.92 (10.71, 20.79).

The overall CHD incidence rate, in our center, was 6.46 per 1000 live births. In the 669 infants born at 30 weeks of gestation or greater, seventy-one infants flagged correctly by NPCU, with ten infants in the *other* group. Diagnoses included PS (50%), ASD (30%) or VSD (10%). In the *other* group, all CHD were classed non-major, with no cardiac interventions needed to date. A further 354 patients, without CHD, were excluded by NPCU (*true negatives*) with 164 patients flagged for cardiology review by NPCU but did not have CHD.

## Discussion

Neonatologists perform cardiac ultrasound (NPCU) is an important assessment tool to assess cardiac hemodynamics in premature and unwell infants. In premature infants < 30 weeks gestation routinely receiving NPCU studies, we identified 109 (5%) with a de novo diagnosis of congenital heart disease (CHD) and 11 with critical or major CHD conditions requiring early cardiac intervention, who would have otherwise been diagnosed later. All patients where there was suspected CHD on NPCU were appropriately referred to cardiology. The NPCU was able to facilitate prompter cardiology referral and assist cardiologists in earlier diagnosis in all cases. No major or critical CHD were missed on NPCU *flagged* cardiac scans. Our study suggests that NPCU, although not intended for this purpose, may detect de novo CHD despite the implementation of modern prenatal imaging and pulse oximetry saturation screening techniques. As such, NPCU-trained clinicians should be adept at recognizing abnormalities in cardiac structures during hemodynamic assessment.

In Australia, training for neonatologists in cardiac ultrasounds is guided by the Australasian Society for Ultrasound in Medicine, resulting in a Certificate of Clinician-Performed Ultrasound; this demonstrates significant hands-on training in functional echocardiography [18]. This training includes overlap with features from both North American and European curriculum [14, 16]. NPCU is not a screening tool for CHD and does not require trainees to exclude CHD [13], our training programs rigorously teaches functional hemodynamic assessments of the heart where anatomical review is required and significant CHD (such as left ventricular outflow obstruction) can be detected [14, 16]. This is partly because appropriate hemodynamic assessment, for example, assessment of a PDA or pulmonary hypertension, is strongly dependent upon anatomical variance and may be limited by the presence of CHD. Effective NPCU services should be able to identify lesions using structured training and appropriate supervision [6, 16]. They should then closely collaborate with cardiology services to ensure optimal follow-up and care [18, 19]. However, in various centers access to cardiology oversight is not possible [20, 21]. If a center is implementing NPCU, adequate attention to training and support is paramount to the reliable detection of CHD and subsequent appropriate hemodynamic assessment [12, 16]. We suggest that NPCU training must include teaching about recognition of early and significant CHD with demonstration of normal cardiac chambers, connections, and valves prior to hemodynamic assessment for clinical decision making [22]. Currently, the degree to which this is practiced varies widely across countries and neonatology centers with focus on standard curriculum and protocols required [20, 23].

CHD continues to be detected by the NPCU despite multiple advances in prenatal and postnatal screening for CHD. In Australia, obstetric ultrasonography has seen recent advances that have contributed to the detection of CHD [24]. NPCU may also be considered within the spectrum of tools available to detect CHD, along with pulse oximetry screening, and are being increasingly used in Australia [8, 10]. Each screening measure, although not designed for this purpose, may opportunistically detect critical CHD despite limitations in detecting certain types of CHD, such as PS [20, 25]. With appropriate training and oversight via pediatric cardiology services, NPCU may improve upon current screening for CHD, almost as an extension of the clinical examination [13, 26]. Other Australian studies have shown de novo CHD diagnosis by NPCU in 14% of CHD cases, but with a vast majority were still diagnosed prenatally [19].

CHD detection, within this study, occurs at similar rates to prior studies but continues to produce a high rate of false positives, due to the need for clinicians to over report CHD based on limitations in training and experience [13, 18]. In our cohort, CHD was diagnosed at a higher rate if flagged during NPCU with early diagnosis being a positive influence on clinical cardiology management. Our focus is less upon the exact CHD diagnosis but rather the prompt recognition of abnormal anatomy, leading to the need for referral. Decreased precision in recognizing more minor anomalies is expected in sonographers with varying experiences and in those with evolving or complex cardiac lesions [27]. There remain many CHD subtypes that may not be apparent on early NPCU, such as PS and ASD, and we expect the rate of detection of these lesions to be lower for NPCU than if the scan was completed later in life. For example, ASDs may continue to be difficult to ascertain from a PFO within early life and PS may progress with chronological age. Errors in detection continue to occur within other screening methods and may be related to sonographer experience, the timing of the scan within the context of chronological age and the weight/gestational age of the baby being scanned [28, 29]. The rate of detection of NPCU continues to be user dependent and we continue to expect that some of these lesions will not be routinely determined on NPCU, as they may not be obvious in the first few days of life when NPCU is routinely performed.

This data is from a tertiary-level NICU with close collaborative ties to pediatric cardiology services and we must acknowledge how this setup varies across the world [16, 20, 21, 23]. In other centers, NPCU may be performed with variable training and protocols without standards that are translatable elsewhere [12, 23, 30]. However, delays in accessing timely cardiology services or the requirement of a neonatal transfers warrants focus on improved CHD detection and management on whatever scan is completed whether it be POCUS, NPE or TnECHO. The accuracy of diagnosis by neonatologists needs to be further explored, and anatomic concordance data is not available for this study because the role of NPCU is not to diagnose and is left to our cardiologists to review scans suspicious for CHD. Without cardiology oversight the successful detection and management of CHD may not always be possible. For example, in many of our NPCU scans hemodynamic assessment was abandoned because of the significant CHD, making assessment inaccurate and requiring an urgent formal echocardiogram. The retrospective nature of the study design is a limitation. Earlier scans were completed by a range of neonatologists, at differing levels of experience and with indications for NPCU being historically driven as opposed to current Australasian training which is becoming more rigid, and guideline driven. We acknowledge that minor CHD may still exist but remain undiagnosed in the true negative group, and that younger members of the cohort may also receive cardiac intervention in the future.

NPCU can accurately identify CHD in infants without a prenatal diagnosis and may lead to earlier identification, referral, and treatment for CHD. The success of the NPCU involves clear and structured guidelines for its performance, appropriate training, accreditation, and supervision, as well as strong collaboration between neonatal and cardiology services. CHD continues to remain within the realm of cardiologists to diagnose and treat. Prospective studies and training developments will assist in appropriate detection of and referral of CHD during neonatologist performed cardiac ultrasound.

## Data Availability

Due to the sensitive nature of the data, access will only be granted upon reasonable request to appropriate ethics departments within WA Health.
